# Carbon-Based Nanomaterials 3.0

**DOI:** 10.3390/ijms23169321

**Published:** 2022-08-18

**Authors:** Ana M. Díez-Pascual

**Affiliations:** Universidad de Alcalá, Facultad de Ciencias, Departamento de Química Analítica, Química Física e Ingeniería Química, Ctra. Madrid-Barcelona Km. 33.6, 28805 Alcalá de Henares, Madrid, Spain; am.diez@uah.es

Carbon-based nanomaterials are currently attracting a lot of interest in many fields, ranging from medicine and biotechnology to electronics, energy storage, and sensing applications [1,2]. They show a variety of shapes, from 0D fullerenes, nanodiamonds, and quantum dots (QDs) to 1D carbon nanotubes (CNTs), and 2D graphene (G) and its derivatives graphene oxide (GO) and reduced graphene oxide (rGO) [3]. Furthermore, new carbon-based nanomaterials are currently under investigation, such as mutated graphene-like nanomaterials, which have been found to be very effective for the removal of organic pollutants from wastewater [4], as well 3D carbon monolithics, which have great potential for the decontamination of radioactive substances [5].

One characteristic of all carbon nanomaterials is the possibility of functionalizing them through non-covalent and covalent methods [6,7], which generally modifies their hydrophilic, electronic, optical, and mechanical properties. Non-covalent approaches are attained via π–π stacking, electrostatic forces, and Van der Waals forces. On the other hand, covalent functionalization can be performed via simple oxidation, leading to oxygen-containing groups suitable for reacting with functional groups of other molecules or polymers. This Special Issue “Carbon-Based Nanomaterials 3.0”, with a collection of 10 original contributions and 2 literature reviews, offers select examples of the surface modifications of carbon nanomaterials that adapt their physicochemical properties, as well as their applications in a variety of fields, such as supercapacitors, sensors, antimicrobial coatings, bioimaging, decontamination, and so forth. 

With an enlarged global focus on tackling the challenges of environmental pollution, the interest in novel energy devices as an alternative to petroleum-based ones has also increased. In this regard, supercapacitors can be designed for use in environmentally friendly vehicles and new renewable energy; however, the limitation of a low energy density remains a challenge [8]. To further increase the power density of supercapacitors, mesoporous carbon nanomaterials can be used. However, activated carbons with high mesopore volumes generated via physical activation are not economically viable. Thus, to develop cost-effective high-performance supercapacitors with high energy and power densities, the preparation of novel mesoporous activated carbons should be investigated. In this regard, Bang et al. [9] have recently synthesized a kenaf-derived activated carbon (KAC) for a high-power density supercapacitor via phosphoric acid activation. Kenaf was chosen as the precursor due to its high productivity, and phosphoric acid activation was applied to create a high specific surface area and advanced mesoporous structure. The pore-growth mechanism for KAC through phosphoric acid activation was explored by analyzing the textural properties and crystal structures. The electrochemical properties of KAC were compared with commercial activated carbon, and an improvement was found both in the specific capacitance and the ion-diffusion resistance. Mesopore control of the electrode material is crucial in improving the supercapacitor resistance and output.

Hierarchical porous activated carbon (HPAC) is another interesting active material for supercapacitors due to its huge specific surface are. Preparing electrodes with high-mass loading is interesting in providing high total capacitances and gravimetric or volumetric energy densities [10]. Therefore, developing an HPAC that increases the mass loading of the resulting electrode is highly desirable. In this regard, Amirtha et al. [11] prepared a novel hydrogel-derived encapsulated H-HPAC (H@H) composite material, with reduced specific surface area and pore parameters but increased proportions of nitrogen species. The free-standing and flexible H@H electrodes showed remarkable reversible capacitance, rate capability, and cycling stability and are also promising electrode materials for other energy storage fields such as metal–ion capacitors. 

In another study, a novel carbonaceous material was prepared from cellulose carbonized via two-steps—hydrothermal and thermal carbonization, in order—without any chemicals [12], giving high yields after a treatment at 600 °C under an inert atmosphere. This led to nanospheres with increased specific surface areas, as confirmed by SEM, FTIR, X-ray diffraction, and Raman spectroscopy, as well as enhanced conductivity. The nanospheres were used as a dispersed phase in electrorheological fluids, displaying exceptional electrorheological effects, considerably surpassing recent state-of-the-art findings. These new carbonaceous particles prepared from renewable cellulose have further potential to be utilized in many other applications that require conducting carbonaceous structures with high specific surface area such as adsorption, catalyst, filtration, and energy storage, to mention but a few.

GO is well known for its outstanding fluorescence quenching capability. Sun et al. [13] prepared a water-soluble positively charged graphene oxide by grafting polyetherimide onto GO nanosheets via a carbodiimide reaction. Compared with conventional GO, the fluorescence quenching ability of the DNA strand of the novel positively charged one was significantly improved via an additional electrostatic interaction. The DNA probe was almost completely quenched for concentrations of the positively charged GO as low as 0.1 µg/mL. This quenching ability was used to develop a sensor for Hg^2+^ detection, leading to a linear concentration range of 0–250 nM, with a limit of detection of 3.93 nM, and it was successfully applied to real samples of pond water, leading to recoveries in the range from 99.6% to 101.1%.

Based on the fluorescence quenching ability of nitrogen- and phosphorous-doped carbon dots, a simple and selective sensor for glutathione detection was also developed [14]. The reductant potential of the doped carbon dots was used to synthesize AuNPs and to subsequently form composites, which were characterized via spectroscopic and microscopic techniques, including electrophoretic light scattering and XRD. The overlap of the fluorescence emission spectrum of the doped carbon dots and the absorption spectrum of AuNPs resulted in an effective inner filter effect in the composite material, leading to a quenching of the fluorescence intensity. In the presence of GSH, the fluorescence intensity of the composite was recovered, leading to a sensing method with a limit of detection of 0.1 µM.

Nitrogen-doped amino acid-functionalized GQDs show enhanced photoluminescence and photostability and lead to the generation of reactive oxygen species through two-photon photodynamic therapy (PDT) [15]. This amino-N-GQDs can be used as two-photon contrast probes to trail and localize analytes in in-depth two-photon imaging executed in a biological environment along with two-photon PDT to eliminate infectious or multidrug-resistant microbes.

Nitrogen oxides (NOx) are amongst the foremost atmospheric pollutants; hence, it is imperative to screen and detect their presence in the atmosphere. For such a purpose, low-dimensional carbon structures have been broadly used as NOx sensors. In particular, CNTs have been applied for sensing toxic gases due to their high specific surface area and excellent mechanical properties. Even though pristine CNTs have shown promising performance for NOx detection, several strategies have been developed such as surface functionalization and defect engineering to expand the NOx-sensing ability of pristine CNT-based sensors. In this regard, the surface modification approaches used in the recent decade to modify the sensitivity and the selectivity of CNTs to NOx have recently been reviewed [16]. 

Other atmospheric contaminants that threaten the environment and life include CO, CO_2_, CH_4_, and O_3_. Canales et al. [17] have explored the use of small fullerenes such as C_30_ for the adsorption of these pollutants. They performed computational simulations to investigate their adsorption on graphene-semifullerene (C_30_) surfaces, considering two C_30_ geometries—hexagonal and pentagonal bases—and found that it is possible to dope all surfaces with Li, Ti, and Pt, which can be used as effective catalysts.

On the other hand, given that the control over radioactive species is currently critical, the development of functional materials for the decontamination of radioactive substances has also become imperative. In this regard, Bae and coworkers [5] have recently developed a 3D porous carbon monolith functionalized with Prussian blue particles via the removal of colloidal silica particles from exfoliated graphene/silica composite precursors. The colloidal silica acted as a template and provided enough surface area that could accommodate potentially hazardous radioactive substances by adsorption. The exceptional surface and pore structure of the novel carbon monolith was examined using SEM, XRD, FTIR, and XPS analysis. Moreover, a nitrogen adsorption/desorption study showed that surface area and pore volume increased significantly compared with the starting precursor. It was found that the novel nanomaterial had a higher adsorption capacity than that of pristine porous carbon monoliths to most radioactive ions and, hence, can be used for decontamination in many fields.

Studying acoustic plasmons (APs) in single-layer, double-layer, and multilayer graphene or in metal/dielectric/graphene superstructures is another active field of research. Although the mechanism of the formation of these plasmons in electrostatically biased graphene or at noble metal surfaces is well known, the mechanism of their formation in alkali-doped graphene is not well understood yet. In this regard, Marušic and coworkers have investigated the interplay of the p and s intraband transitions with plasmon resonance [18]. Their work illustrates the importance of understanding the nature of the chemical bonding between alkaline atoms and graphene and the perpendicular dispersivity of the dynamical response in theoretical simulations of low-energy plasmons.

On the other hand, the interaction between photons and polarization modes can result in the formation of hybrid photon polarization modes, called polaritons. The same authors [19] have shown that 2D layered nanomaterials enable the formation of well-defined exciton–polaritons even at room temperature and that the exciton–photon coupling can be manipulated simply by changing the number of single layers. These nanostructures can be applied in photonic devices, such as LED, telecommunications, or chemical and biological sensing.

As known, graphene is a versatile compound with many outstanding properties, providing a combination of a huge surface area, a high strength, and thermal and electrical properties, with a wide array of functionalization possibilities. However, the available literature on graphene-based coatings in dentistry and medical implant technology is scarce. Srimaneepong and coworkers [20] have recently provided a comprehensive and well-organized review on graphene applications in such field. Graphene displays good biocompatibility, corrosion prevention, and excellent antimicrobial properties to prevent the colonization of bacteria. Moreover, graphene coatings improve cell adhesion and osteogenic differentiation, and promote antibacterial activity to parts of titanium unaffected by the thermal treatment. Additionally, the coating can improve the surface properties of implants, which can then be used for biomedical applications. Hence, graphene and its derivatives may hold the key to the next revolution in dental and medical technology.
